# Persistent Secondary Hypogonadism Following Chronic Opioid and Anabolic Steroid Exposure: A Case Report of Long-Term Testosterone Replacement Therapy and Its Complications

**DOI:** 10.7759/cureus.114397

**Published:** 2026-08-12

**Authors:** Elaina Hollis, Robert Neamtu, Andrew Sandoval, Karen Bruce

**Affiliations:** 1 Family Medicine, William Carey University College of Osteopathic Medicine, Hattiesburg, USA; 2 Family Medicine, Forrest General Family Medicine Residency Clinic, Hattiesburg, USA

**Keywords:** bilateral gynecomastia, chronic opioid use, opioid-induced androgen deficiency, secondary hypogonadism, testosterone replacement therapy (trt)

## Abstract

Secondary hypogonadism is a clinically significant consequence of hypothalamic-pituitary-gonadal (HPG) axis suppression and may result from multiple exposures, including chronic opioid and anabolic steroid use. Long-term treatment strategies for persistent secondary hypogonadism, particularly testosterone replacement therapy (TRT) and its complications, remain incompletely characterized. This case describes a male patient with a history of both opioid and anabolic steroid exposure who developed persistent acquired secondary hypogonadism, with potential contributions from both opioid-induced androgen deficiency (OPIAD) and anabolic steroid-induced hypogonadism (ASIH). The patient subsequently underwent approximately 13 years of TRT, with sustained symptomatic benefit but clinically significant complications including erythrocytosis and late-onset gynecomastia. At the time gynecomastia was documented, serum estradiol testing and breast imaging were not available in the records reviewed, and the diagnosis was based primarily on patient-reported symptoms. This case highlights the challenges of determining the etiology of persistent HPG axis suppression following multiple suppressive exposures and emphasizes the importance of longitudinal monitoring for complications associated with prolonged TRT.

## Introduction

Opioid-induced androgen deficiency (OPIAD) is a frequently underrecognized consequence of chronic opioid exposure, arising from suppression of the hypothalamic-pituitary-gonadal (HPG) axis and resulting in secondary hypogonadism. Anabolic-androgenic steroid exposure can similarly suppress the HPG axis, creating diagnostic uncertainty in patients with histories of both substance exposures. Despite the significant impact of secondary hypogonadism on the quality of life, including diminished libido, fatigue, and impaired motivation, its long-term clinical course and optimal management in patients with multiple contributing factors remain insufficiently defined. Testosterone replacement therapy (TRT) is commonly utilized to mitigate the symptoms of hypogonadism; however, its prolonged use introduces additional risks and complexities that are not well characterized. We report the case of a male patient with a history of chronic opioid and anabolic steroid use who developed persistent acquired secondary hypogonadism, with potential contributions from both opioid-induced androgen deficiency (OPIAD) and anabolic steroid-induced hypogonadism (ASIH). This case is notable for a 13-year course of TRT, during which the patient experienced sustained symptomatic benefit alongside clinically significant complications, including erythrocytosis and gynecomastia. This report offers longitudinal insight into persistent HPG axis dysfunction following multiple suppressive exposures, highlights the underrecognized sequelae of chronic testosterone therapy, and underscores the challenges of establishing causality and managing long-term secondary hypogonadism in patients with multifactorial risk factors [1].

## Case presentation

The patient initially presented with decreased libido, low energy, and decreased motivation. Available laboratory records demonstrated low total testosterone beginning in October 2012 (244 ng/dL), with a subsequent level of 239 ng/dL in January 2013. Further evaluation demonstrated low free testosterone and suppressed gonadotropins, consistent with hypogonadotropic hypogonadism. Brain MRI was normal, and semen analysis demonstrated azoospermia.

**Figure 1:**
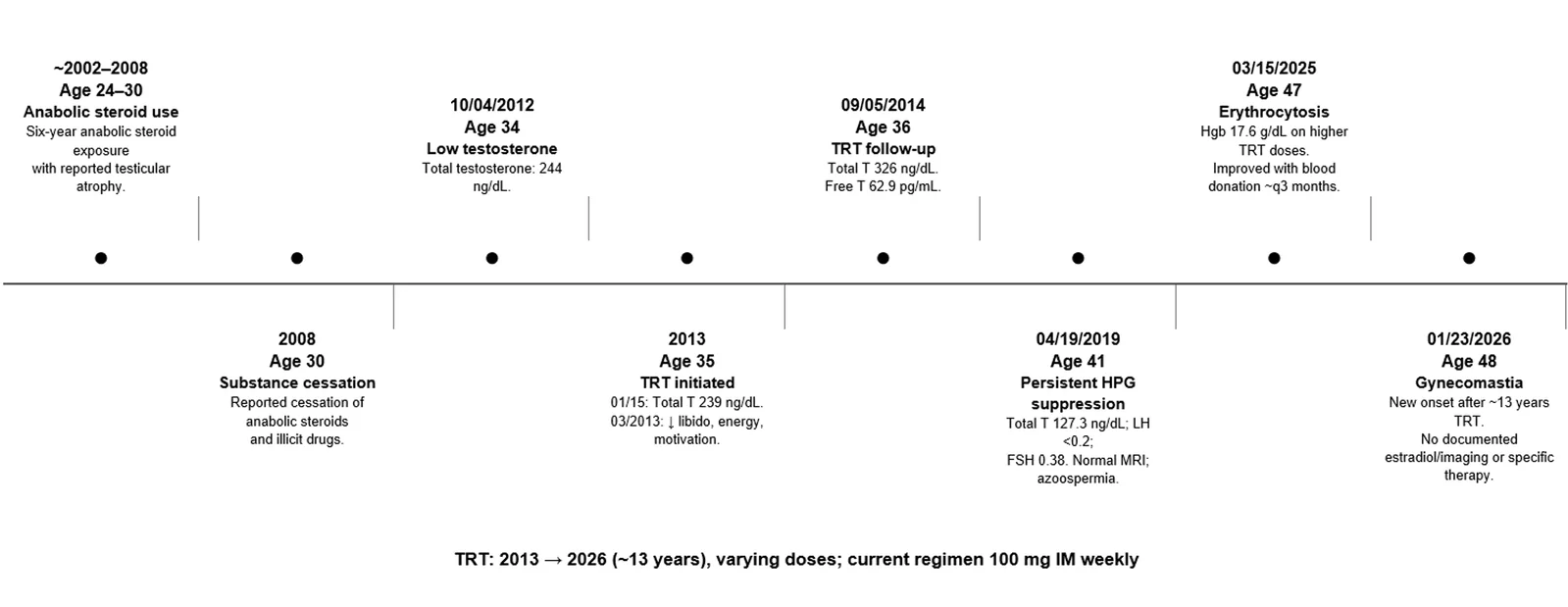
Visual Timeline of Opioid and Anabolic Steroid Exposure, Secondary Hypogonadism, Testosterone Replacement Therapy, and Treatment-Related Complications Abbreviations: ASIH, anabolic steroid-induced hypogonadism; FSH, follicle-stimulating hormone; HPG, hypothalamic-pituitary-gonadal; IM, intramuscular; LH, luteinizing hormone; OPIAD, opioid-induced androgen deficiency; T, testosterone; TRT, testosterone replacement therapy.

His history was significant for approximately six years of prior anabolic steroid use, during which he reported testicular atrophy, as well as prior intravenous oxycodone and morphine use and recreational methamphetamine use. Importantly, the patient reported sustained sobriety from illicit drugs since 2008, preceding the first documented low testosterone level in 2012. Given his substantial history of both opioid and anabolic steroid exposure, a single primary etiology for persistent HPG axis suppression could not be definitively established. His acquired secondary hypogonadism was therefore considered multifactorial, with potential contributions from both OPIAD and ASIH. The chronology of the patient’s substance exposures, development of hypogonadism, TRT course, and subsequent treatment-related complications is summarized in Figure 1.

The patient’s clinical course demonstrates biochemical evidence of hypogonadism following reported cessation of prior substance exposures, with continued hormonal abnormalities documented during his subsequent treatment course. Serial laboratory testing documented persistently abnormal testosterone levels and later demonstrated marked suppression of gonadotropins, supporting ongoing HPG axis dysfunction. Available longitudinal hormonal and hematologic laboratory findings are summarized in Table 1.

**Table 1:** Longitudinal Laboratory Findings During the Clinical Course

Laboratory Test	Patient Value	Reference Range	Interpretation
Total Testosterone (10/04/2012)	244 ng/dL	300-1,000 ng/dL	Low
Total Testosterone (01/15/2013)	239 ng/dL	300-1,000 ng/dL	Low
Total Testosterone (09/05/2014)	326 ng/dL	300-1,000 ng/dL	Within reference range
Free Testosterone (09/05/2014)	62.9 pg/mL	66-220 pg/mL	Low
Total Testosterone (04/19/2019)	127.3 ng/dL	300-1,000 ng/dL	Low
Luteinizing Hormone (LH) (04/19/2019)	<0.2 mIU/mL	1.7-8.6 mIU/mL	Low
Follicle-Stimulating Hormone (FSH) (04/19/2019)	0.38 mIU/mL	1.5-12.4 mIU/mL	Low
Hemoglobin (03/15/2025)	17.6 g/dL	13.5-17.5 g/dL	Elevated

Additional historical laboratory values, including baseline prolactin, thyroid studies, iron studies/ferritin, baseline hematocrit, and serum estradiol levels, were not available in the records reviewed.

Treatment consisted of testosterone replacement therapy (TRT) at varying doses over approximately 13 years. The patient currently receives a 100 mg intramuscular injection weekly and reports improvement in libido, energy, and motivation with treatment. He has experienced fluctuations in symptoms toward the end of his dosing intervals, including decreased concentration, appetite, mood, and libido prior to his next injection, consistent with symptomatic trough effects associated with weekly intramuscular administration.

Long-term TRT was complicated by erythrocytosis, nodular acne, and, most recently, gynecomastia. In March 2025, hemoglobin reached 17.6 g/dL while the patient was receiving higher testosterone doses. The erythrocytosis subsequently improved with blood donation approximately every three months. In January 2026, after approximately 13 years of TRT, the patient reported new-onset gynecomastia and inquired about an over-the-counter supplement for symptom management. Available records did not document serum estradiol testing, breast imaging, initiation of a selective estrogen receptor modulator (SERM) or aromatase inhibitor, adjustment of TRT specifically for gynecomastia, or surgical referral.

## Discussion

OPIAD is a common, yet underdiagnosed, result of chronic opioid use. The signs and symptoms of OPIAD include but are not limited to decreased libido, weight gain, depression, and decreased energy, which can significantly impact the quality of life [1]. Given the widespread use of opioids in pain management, the clinical implications of OPIAD extend beyond individual cases and reflect a broader public health concern.

Opioids remain an important treatment for acute and chronic pain, although chronic exposure is associated with numerous adverse effects. Current Centers for Disease Control and Prevention (CDC) guidance emphasizes individualized assessment of the benefits and risks of opioid therapy, appropriate dosing and duration, and consideration of tapering or discontinuation when treatment risks outweigh benefits [2]. Among the less frequently recognized consequences of chronic opioid exposure are endocrine abnormalities, including suppression of the hypothalamic-pituitary-gonadal (HPG) axis.

While opioid dependence and withdrawal are well-recognized complications of long-term opioid therapy, the systemic endocrine sequelae are often underrecognized. Specifically, prolonged opioid use disrupts neuroendocrine signaling and suppresses the hypothalamic-pituitary-gonadal (HPG) axis. Mechanistically, opioids inhibit gonadotropin-releasing hormone (GnRH) secretion from the hypothalamus, resulting in decreased luteinizing hormone (LH) and follicle-stimulating hormone (FSH) release from the pituitary gland and subsequent reduction in testosterone and estrogen production by the gonads. Opiates may also decrease adrenal androgen synthesis and directly inhibit testicular testosterone synthesis [1]. This mechanism of HPG axis suppression is what underscores secondary hypogonadism, with chronic opioid use being one acquired etiology.

In this patient, however, opioid exposure cannot be considered the sole potential cause of persistent HPG axis dysfunction. Anabolic-androgenic steroid exposure can similarly suppress the HPG axis through negative feedback, reducing endogenous gonadotropin and testosterone production. Recovery following anabolic steroid cessation is variable, with testosterone recovery generally occurring over months and gonadotropin recovery typically occurring within several months, although recovery may be prolonged depending on factors such as age and the extent of prior androgen exposure [3]. Given this patient’s six-year history of anabolic steroid use with reported testicular atrophy, both ASIH and OPIAD are plausible contributors to his subsequent hypogonadotropic hypogonadism. Therefore, his persistent secondary hypogonadism is more appropriately considered multifactorial rather than attributed exclusively to prior opioid exposure.

Secondary hypogonadism can significantly impact the quality of life for both men and women. Common symptoms in both sexes include infertility, loss of libido, low energy levels, depression, and anxiety. In men, there can be impotence, erectile dysfunction, and loss of muscle mass and strength. In women, irregular menstrual periods and amenorrhea are common. The reported prevalence of opioid-induced hypogonadism varies widely, ranging from 21% to 86% [4]. The variability in reported prevalence may in part reflect differences in opioid formulation and route of administration.

Evidence suggests that the route of opioid administration influences both the degree of hormonal suppression and symptom severity. In a prospective study evaluating intrathecal opioid therapy, serum testosterone levels declined significantly from 7.7±1.2 nmol/L to 2.0±0.7, 2.8±0.5, and 4.0±0.9 nmol/L at 1, 4, and 12 weeks, respectively (P<.0001), demonstrating rapid and profound suppression of the hypothalamic-pituitary-gonadal axis [4]. Notably, intrathecal administration appeared to affect men and women relatively equally.

In contrast, chronic oral opioid therapy has been associated with greater sex-based disparities in hypogonadism prevalence [5]. A cross-sectional study of 26 patients receiving long-term oral opioids reported hypogonadism in 75% of men compared with 21% of women, suggesting a disproportionately higher risk of gonadal suppression among males. Additionally, osteopenia was observed in approximately 50% of men compared with 21% of women after at least one year of therapy, although this difference did not reach statistical significance [6]. Interpretation of these findings is limited by small sample size and longer opioid exposure among male participants. However, the trend potentially supports a clinically meaningful sex-based predisposition to opioid-induced hypogonadism.

Further supporting these findings, an observational study of 54 men receiving chronic oral opioids demonstrated that 89% exhibited decreased concentrations of free testosterone, estradiol, dihydrotestosterone, luteinizing hormone, and follicle-stimulating hormone. Although all participants reported normal erectile function prior to opioid initiation, 87% subsequently developed severe erectile dysfunction and decreased libido, highlighting the functional consequences of opioid-induced hormonal suppression [4].

Opiate-induced hypogonadism is more common than historically thought and remains a challenge to diagnose. Notably, many symptoms of androgen deficiency (fatigue, low libido, mood changes) overlap with those experienced by patients with chronic pain, the primary indication for opioid therapy [1]. Additionally, there are no established guidelines for the management of OPIAD. Thus, clinicians may miss the diagnosis or manage the condition inconsistently. 

For the diagnosis of OPIAD in men, it is reasonable to begin by measuring morning serum testosterone levels, a normal range falling between 300 to 800 ng/dL. If the value returns abnormal, then further confirmatory tests such as free testosterone, serum hormone binding globulin (SHBG), LH, FSH, and prolactin should be obtained. Unfortunately, there are limited diagnostic criteria for women with OPIAD, and those with suspected OPIAD, especially those who must remain on opioid therapy, should be referred to an endocrinologist [1]. 

Management of OPIAD in men includes opioid tapering or discontinuation when clinically feasible, as well as consideration of testosterone replacement therapy. Evidence suggests that cessation of opioid use may result in recovery of endogenous testosterone levels following discontinuation [1]. Accordingly, symptoms of OPIAD may improve following opioid cessation. However, in the present case, hypogonadism persisted following reported cessation of both opioid and anabolic steroid exposure. Because both substances can suppress the HPG axis, the relative contribution of each exposure to persistent dysfunction cannot be determined retrospectively. This diagnostic uncertainty highlights the difficulty of attributing persistent secondary hypogonadism to a single etiology in patients with multiple prior HPG axis-suppressive exposures.

For men with symptomatic opioid-associated hypogonadism who require ongoing opioid therapy, testosterone replacement therapy (TRT) may be considered when clinically appropriate. TRT may also be considered in patients with persistent symptomatic hypogonadism following opioid discontinuation. TRT can be administered through several exogenous formulations, including intramuscular injections, transdermal gels or patches, buccal systems, intranasal preparations, and subdermal pellets. Intramuscular testosterone is commonly utilized, with standard dosing regimens of 75-100 mg weekly or 150-200 mg every two weeks. The therapeutic goal is to restore serum testosterone concentrations to the physiologic range and improve clinical symptoms in hypogonadal men [7].

A randomized study evaluated graded weekly doses of testosterone (25, 50, 125, 300, and 600 mg) to assess dose-response relationships with serum testosterone levels, muscle mass, strength, and adverse effects [8]. A significant dose-dependent increase in fat-free mass was observed in men receiving 125, 300, and 600 mg weekly (+3.4, +5.2, and +7.9 kg, respectively), with changes strongly correlated with testosterone dose (P=.0001). Improvements in leg press strength, leg power, thigh and quadriceps muscle volume, hemoglobin, and insulin-like growth factor 1 (IGF-1) were positively associated with increasing testosterone concentrations. In contrast, high-density lipoprotein (HDL) cholesterol levels were inversely correlated. Although reductions in HDL suggest a potential increase in cardiovascular risk at higher doses, total cholesterol, low-density lipoprotein (LDL) cholesterol, and triglyceride levels did not demonstrate significant changes across dosing groups [8].

Another study demonstrated a significant dose-response relationship between graded doses of testosterone enanthate and serum estradiol levels (P<.001), attributable to increased aromatization of testosterone to estradiol via the aromatase enzyme [9]. Although a clear dose-dependent relationship with gynecomastia incidence has not been definitively established, increased conversion of testosterone to estradiol provides a biologically plausible mechanism for estrogen-mediated breast tissue proliferation during TRT. Aromatase activity may also increase with age and greater adipose tissue mass, potentially altering the androgen-to-estrogen balance over the course of long-term treatment. These factors may contribute to the development of gynecomastia later in the treatment course rather than immediately following TRT initiation. In this patient, however, longitudinal estradiol levels and body composition data were unavailable; therefore, the mechanism underlying the development of gynecomastia after approximately 13 years of TRT cannot be definitively established.

A randomized, double-blind, placebo-controlled trial by Basaria et al. evaluated 84 men (mean age, 49 years) receiving chronic opioid therapy with total testosterone levels <350 ng/dL [10]. Participants were assigned to receive either transdermal testosterone gel or placebo for 14 weeks. Compared with placebo, testosterone therapy resulted in significant improvements in sexual desire and body composition, with no significant differences in self-reported pain levels. These findings suggest that testosterone replacement can be safely administered in patients on chronic opioid therapy without compromising analgesic efficacy. However, the short duration of this study limits assessment of long-term adverse effects, including gynecomastia and other estrogen-mediated complications [10].

Rabkin et al conducted a six-week randomized, double-blind, placebo-controlled trial involving 74 hypogonadal men with human immunodeficiency virus (HIV) [11]. Patients receiving testosterone therapy demonstrated significantly greater improvements in libido, with 74% (28/38) reporting being “much or very much improved” compared with 19% (6/32) in the placebo group (P<.001). Additionally, 59% of men in the testosterone group reported improved energy levels compared with 25% in the placebo group (P<.01), and 58% reported improved mood compared with 14% in the placebo group (P=.08). These findings further support the symptomatic benefits of testosterone therapy in hypogonadal populations [11].

Wu et al. evaluated 271 men (mean age, 31.8 years) receiving testosterone enanthate to assess potential adverse effects [12]. Gynecomastia developed in 24 participants, while nine men (one Chinese, eight non-Chinese) experienced prostate-related complications. The most frequently reported adverse effects were non-estrogenic and included injection site pain, acne, fatigue, and weight gain [12].

Another study investigated the use of aromatase inhibitors, specifically anastrozole (0.5 mg three times weekly), in men undergoing testosterone replacement therapy with elevated estradiol levels (>60 pg/mL regardless of symptoms, or 40-60 pg/mL with associated symptoms)[13]. Median estradiol levels decreased significantly from 65 pg/mL before treatment to 22 pg/mL after treatment (P<.001), demonstrating the efficacy of aromatase inhibition in reducing circulating estradiol. However, the study did not demonstrate that anastrozole was effective in reversing established glandular gynecomastia or in preventing gynecomastia in asymptomatic individuals [13].

However, in a case series by Rhoden and Morgentaler, two hypogonadal men undergoing testosterone replacement therapy developed gynecomastia that was successfully treated with the aromatase inhibitor anastrozole [14]. In both cases, initiation of anastrozole led to the resolution of breast enlargement and symptoms, and testosterone therapy was able to be continued without recurrence of gynecomastia on follow-up. These findings highlight the role of aromatase inhibition in reducing estradiol-mediated breast tissue proliferation and managing testosterone-induced gynecomastia. This suggests that aromatase inhibitors could be a less invasive potential treatment option for gynecomastia for patients who are unwilling to go through radiation or surgical treatment. More research is needed on this treatment modality compared to the current gold standard options.

While aromatase inhibitors may be beneficial in select cases, broader evidence supports other treatment modalities as more effective. In a clinical review by Johnson et al., selective estrogen receptor modulators (SERMs), particularly tamoxifen, were identified as the most effective pharmacologic therapy for reducing gynecomastia and associated symptoms, demonstrating superiority over both aromatase inhibitors and radiotherapy [15]. However, the quality of evidence supporting pharmacologic therapies remains limited, consisting largely of small, randomized trials and case series. In contrast, surgical correction is considered the gold-standard treatment for persistent or symptomatic gynecomastia, providing reliable cosmetic and symptomatic improvement, although this recommendation is also primarily supported by observational data rather than high-quality randomized trials [15]. These findings suggest that while SERMs represent the most effective medical therapy, overall treatment recommendations are constrained by a relative lack of robust, high-level evidence. In the present case, available records did not document serum estradiol testing, breast imaging, adjustment of TRT specifically for gynecomastia, initiation of an aromatase inhibitor or SERM, or referral for surgical management. Therefore, although several therapeutic approaches have been described for TRT-associated gynecomastia, the effectiveness of these strategies cannot be evaluated in this patient. The absence of longitudinal estradiol measurements also limits assessment of whether increased aromatization contributed to his late-onset gynecomastia.

## Conclusions

This case underscores the potential for persistent hypothalamic-pituitary-gonadal axis dysfunction following multiple suppressive exposures, including chronic opioid and anabolic steroid use. Given the patient’s history, the relative contributions of OPIAD and ASIH to persistent secondary hypogonadism cannot be definitively distinguished. Although testosterone replacement therapy provided sustained symptomatic benefit, long-term treatment was associated with clinically significant adverse effects, including erythrocytosis and gynecomastia, necessitating continued monitoring and management. This case highlights the importance of recognizing multifactorial causes of secondary hypogonadism, carefully assessing the long-term risks and benefits of testosterone therapy, and obtaining longitudinal hormonal and hematologic monitoring in patients requiring prolonged treatment.
